# Spontaneous Tumor Lysis Syndrome in the Setting of B-Cell Lymphoma

**DOI:** 10.1155/2010/610969

**Published:** 2010-03-10

**Authors:** Mateusz Opyrchal, Travis Figanbaum, Amit Ghosh, Vincent Rajkumar, Sean Caples

**Affiliations:** Mayo Clinic, Rochester, Minnesota, USA

## Abstract

Tumor lysis syndrome (TLS) presenting in absence of chemotherapy is a rare occurrence. One of the true oncological emergencies, it can lead to significant morbidity and mortality. TLS is a phenomena usually associated with tumor cell death after treatment. The etiology of the spontaneous TLS is not well understood, which complicates the diagnosis. TLS is well known to oncologists but physicians outside of this specialty have little or no experience with this condition. Early recognition and treatment are the keys to limiting the sequela of the condition. Spontaneous tumor lysis syndrome is rare but presents added risks to the patient because of the potential for delayed diagnosis and no benefit of pretreatment. Diagnosis may be further delayed because this may be the first symptom of underlying malignancy. Therefore, it is imperative that all clinicians are familiar with the syndrome to assure timely recognition.

## 1. Introduction

Tumor lysis syndrome (TLS) is a phenomena usually associated with tumor cell death after treatment. The dying cells release vast amounts of electrolytes, protein and nucleic acids into the extracellular space, which can overwhelm homeostatic mechanisms. It may present with multiple electrolyte abnormalities including hyperuricemia, hyperphosphatemia, hypocalcemia and hyperkalemia and can lead to multi-organ dysfunction, affecting the kidneys, heart, skeletal muscle and nervous system; TLS can be fatal. TLS is considered one of the oncological emergencies. Spontaneous TLS, occurring in the absence of chemotherapy, is rare but might portend a worse prognosis. We present a case of spontaneous TLS in a female with a later diagnosis of large B-cell lymphoma.

## 2. Case Presentation

**Figure 1:**
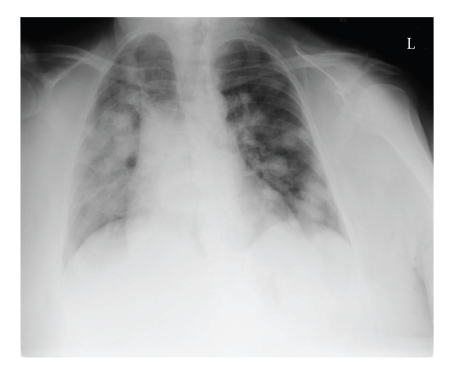
Chest X-ray showing multiple pulmonary nodules.

**Figure 2:**
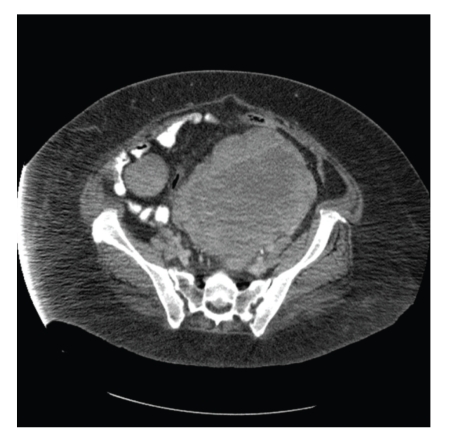
CT of the pelvis with large pelvic mass.

An 82-year-old woman presented to an outside institution with several weeks of increasing dyspnea on exertion, fatigue and weight loss. She was found to be anemic and transfused RBC. Chest X-ray showed bilateral pulmonary nodules (Figure 1) worrisome for metastatic disease. CT of the pelvis and abdomen showed a 16 cm adherent pelvic mass and sizable masses in the gastro-hepatic ligament, adrenal gland, and para-aortic area. (Figure 2) She was referred to our institution. Vital signs revealed a blood pressure of 94/57, pulse 75/min, respiratory rate of 18/min, temperature 36.6°C. Physical examination showed a fatigued appearing woman. The abdomen was obese without palpable mass or splenomegaly. No peripheral lymphadenopathy was palpable; there was 1+ lower extremity pitting edema. Laboratory work showed serum urea nitrogen (BUN) 68 (normal 6–21 mg/dL), potassium 5.2 (normal 3.6–5.2 mmol/L), sedimentation rate 101 f(normal 0–21 mm/1 hr), lactate dehydrogenase (LDH) 478 (normal 122–222 U/L) with additional results summarized in Table 1. 

The patient was admitted for IVF hydration for presumed spontaneous TLS complicated by nonoliguric renal failure. Obstructive uropathy and kidney infiltration by tumor were excluded by CT scan. She was treated with aggressive intravenous hydration and furosemide to maintain urinary output > 200 mL/h. Allopurinol was initiated. Her laboratory values improved over the next 24 hours (Table 1). Other causes of renal failure such as dehydration, contrast nephropathy, vasculitis, and cryoglobulinemic glomerulonephritis were also considered but the initial presentation as well as improvement with therapy confirmed the diagnosis of spontaneous TLS. Fine needle biopsy of the abdominal mass revealed diffuse large b-cell lymphoma, ultimately found to be stage IV-B by PET imaging. 

The patient subsequently underwent chemotherapy with rituximab, doxorubicin, vincristine and prednisone (R-CHOP). At the time of initiation of her chemotherapy her uric acid was back to normal but because of the large tumor burden and the history of spontaneous TLS the decision was made to administer rasburicase one day prior to initiation of chemotherapy to prevent occurrence of acute TLS. She tolerated the treatment well with marked reduction in tumor burden on follow up imaging. There was no recurrence of TLS. Her course was complicated by the development of asymptomatic pulmonary emboli but she completed 6 cycles of R-CHOP therapy with complete tumor remission. Unfortunately one year later she was found to have metastatic thyroid cancer and she passed away shortly after.

## 3. Discussion

TLS is a well-known phenomenon to the oncologist and it most frequently occurs after the initiation of therapy for various malignancies. The risk factors for developing acute TLS after initiation of chemotherapy in all tumors are uric acid level > 7.5 mg/dL at initiation of treatment, underlying renal insufficiency, creatinine > 1.6 mg/dL, hypercalcemia, leukocytosis > 50,000 m^3^, bulky disease, high LDH, high tumor growth fraction [1, 2]. Non-Hodgkin's lymphoma, Burkitt's lymphoma, other aggressive B-cell lymphomas, acute lymphoblastic lymphoma and other hematological malignancies are associated with higher risk of the occurrence of the acute TLS [3, 4]. It can also occur in solid cancers [5]. Spontaneous TLS is a very rare occurrence, which has also been described with both hematological and solid malignancies [5–8]. 

The staple of TLS treatment is prevention. Unfortunately, this is unavailable when presented with a case of spontaneous TLS. The first step in managing hyperuricemia is adequate hydration. Patients should receive 2 to 4 times the daily fluid maintenance unless there are contraindications to large fluid volumes from other comorbidities. The increased urine output should help with excretions of uric acid and phosphate [9, 10]. The general consensus is that the urine output should be above 100 mL/m2/h [10, 11]. If the urine output is still inadequate despite hydration, the use of diuretics is then required. If urine output is still inadequate despite hydration, diuretics should be used (loop diuretics such as furosemide are first-line; osmotic agents such as mannitol have also been utilized). Recent expert guidelines have recommended against alkalinization of urine due to inadequate data on benefits and potential complications from the therapy [10, 11]. 

A recent addition to the armamentarium for treatment of hyperuricemia in TLS is recombinant urate oxidase/rasburicase. As seen in our case, urate oxidase is effective for prevention of TLS in high-risk patients prior to initiation of therapy. It is contraindicated in patients with Glucose-6-phosphate dehydrogenase (G6PD) deficiency, therefore testing, including RBC NADPH, is recommended before its use. Although in the US rasburicase is only approved for use in children, it has shown efficacy in adults as well [12]. Allopurinol can also be used, but it has major limitations. First, it only blocks the production of new uric acid without any effect on uric acid already in circulation. It can also cause xanthine nephropathy. In addition, because it is renally cleared, clinicians must use allopurinol carefully in those with impaired renal function, as is often encountered in this patient population. 

In conjunction with above treatments, patients' electrolytes have to be monitored closely and corrected as abnormalities present themselves. Although the incidence of patients requiring dialysis has decreased, up to 5% of all patients diagnosed with TLS still need the procedure [12]. Therefore, it is always important to involve renal specialist early in the care.

The etiology of the spontaneous TLS is unclear at this point. There are various hypotheses including increased production of glucocorticoids and hyperthermia which lead to increased tumor cell death [6, 13]. It is possible that with the wide spectrum of tumors there can be multiple causes of this syndrome. More research needs to be done in order to better understand causes of spontaneous TLS. Fortunately, spontaneous TLS is rare but there remains the possibility of worse clinical outcomes because of the lack of benefit of pre-treatment. Also, because these patients are often not under the care of oncologists, the syndrome may go unrecognized, which delays appropriate treatment. Therefore, it is imperative that general clinicians are able to recognize the syndrome and initiate adequate care.

## Figures and Tables

**Table 1:** Essential laboratory values during the first 48 hours of treatment.

Time (hrs)	Initial	2	4	7	12	24	48	Normal values
Calcium (Ionized)	4.41	4.09	4.25	4.01	4.25	4.25	4.85	4.80–5.70 mg/dL
Phosphate	5.5	4.5	4.2	4.3	4.4	4.4	4.4	2.5–4.5 mg/dL
Uric Acid	15.6	13.3	12.5	11.8	10.7	9.6	4.2	2.7–6.1 mg/dL
Creatinine	1.8	1.3	1.2	1.1	1	0.9	0.9	0.6–1.1 mg/dL
